# Expression and clinical significance of *IL7R*, *NFATc2*, and *RNF213* in familial and sporadic multiple sclerosis

**DOI:** 10.1038/s41598-021-98691-5

**Published:** 2021-09-28

**Authors:** Seyedeh Zahra Hosseini Imani, Zohreh Hojati, Sheyda Khalilian, Fariba Dehghanian, Majid Kheirollahi, Mehdi Khorrami, Vahid Shaygannejad, Omid Mirmosayyeb

**Affiliations:** 1 Division of Genetics, Department of Cell and Molecular Biology and Microbiology, Faculty of Biological Sciences and Technologies, University of Isfahan, Isfahan, 81746-73441 Iran; 2 Department of Genetics and Molecular Biology, Research Institute for Primordial Prevention of Non-Communicable Disease, School of Medicine, Isfahan University of Medical Sciences, Isfahan, Iran; 3 Isfahan Neuroscience Research Center, Isfahan University of Medical Sciences, Isfahan, Iran

**Keywords:** Gene expression analysis, Genetic techniques, Neurological disorders, Biological techniques, Genetics, Molecular biology, Neuroscience, Biomarkers, Diseases, Neurology

## Abstract

Multiple sclerosis (MS) is a chronic inflammatory and autoimmune disorder of the central nervous system characterized by myelin loss and axonal dysfunction. Increased production of inflammatory factors such as cytokines has been implicated in axon destruction. In the present study, we compared the expression level of *IL7R*, *NFATc2*, and *RNF213* genes in the peripheral blood of 72 MS patients (37 familial MS, 35 sporadic MS) and 74 healthy controls (34 individuals with a family history of the disease, 40 healthy controls without a family history) via Real-time PCR. Our results showed that the expression level of *IL7R* was decreased in the sporadic patients in comparison with other groups. Additionally, there was an increased *NFATc2* expression level in MS patients versus healthy controls. Increased expression of *NFATc2* in sporadic and familial groups compared to the controls, and familial group versus FDR was also seen. Our results also represented an increased expression level of *RNF213* in familial patients as compared to the control group. The similar *RNF213* expression between sporadic and control group, as well as FDR and familial group was also seen. Diagnostic evaluation was performed by receiver operating characteristic (ROC) curve analysis and area under the curve (AUC) calculation. The correlation of clinical parameters including onset age and Expanded Disability Status Scale (EDSS) with our gene expression levels were also assessed. Overall, decreased expression level of *IL7R* in the sporadic cases and increased expression level of *NFATc2* may be associated with the pathogenesis of MS disease. Confirmation of the effects of differential expression of *RNF213* gene requires further studies in the wider statistical populations.

## Introduction

Multiple Sclerosis (MS) is a chronic inflammatory autoimmune disorder of the central nervous system (CNS) affecting about 2–3 million people worldwide^1^. The main causes of the disease are a breakdown of the blood–brain barrier, gliosis of astrocytes, loss of oligodendrocytes and destruction of axons^2^. In all of these stages, pro-inflammatory cytokines could cause or exacerbate the disease compromising the blood–brain barrier. Therefore, inflammation plays a key role in the pathogenesis of the disease^3^. Many of the cells in the innate and adaptive immune system respond to inflammation created within the central nervous system^4^. Mounting criteria have suggested that the major cause of MS is a combination of both environmental and genetic factors^5–7^. Genetic susceptibility plays an important role in the development of the disease, as more than 200 genetic variants associated with MS risk is identified through genome-wide association studies (GWAS) in the last few years^8^. The most susceptible locus associated with this disease is the HLA class Π genes^9^. Mostly, the strongest MS risk factor is human leukocyte antigen (HLA)-DRB^*^15: 01, a major histocompatibility complex (MHC) class Π allele^10,11^. The importance of non-HLA genes in MS has also been repeatedly mentioned. One of the most non-HLA genes was recognized as the Interleukin 7 Receptor (*IL7R*), which is translated to a functional receptor for interleukin 7 (IL7)^12^. Generally, IL7R can be recognized on the surface of the mature T cells and immature B cells. IL7 has a range of biological activities and is essential for the survival, proliferation, and homeostasis of T-cells. It is also a factor in bone marrow culture that can induce the proliferation of B progenitor cells^13^. IL7R may also have a primary signaling function through the autoimmunity cascade. This gene may also be involved in the remyelination process. Deletion of *IL7R* delays the maturation and differentiation of oligodendrocyte cells^14^. Previous studies have shown the association between *IL7R* and MS, and also the link between the *IL7R* haplotypes with the disease has been investigated^15^.

The Wnt signaling pathway is one of the key cascades in regulating the evolution and stability of the immune and blood cells. Although the disruption of this pathway is associated with many pathological conditions, its exact role is still controversial. This pathway appears to regulate several biological processes, including cell proliferation, migration, polarity, differentiation, and axonal growth. Wnt proteins have been shown to regulate T-cell growth and maturation of dendritic cells and also play a significant function in the expression of inflammatory mediators^16^. *NFATc2* is one of the important genes within the Wnt signaling pathway that plays an important role in immune system inflammation. It is one of the several transcription factors regulated by Calcineurin in the Wnt/Ca^2+^ signaling pathway. *NFATc2* also mediates oligodendrocyte differentiation, T-cell function and coordinates the induction of cytokines and immunomodulatory molecules. NFAT proteins can regulate the expression of genes involved in the differentiation of T cell receptors and T helper cells. It is reported that the extracellular calcium influx through cation channels could lead to the increased expression of NFAT target genes^17^. RNF213 is an ATPase and E3 ubiquitin ligase protein, which is involved in the angiogenesis, and degradation of FLNA and NFATc2, that leads to the inhibition of Wnt/Ca^2+^ pathway. Consequently, *RNF213* deficiency has been shown to cause an increased expression of NFAT target genes^18^. Interestingly, these genes play an important role in chronic inflammation, nerve cell destruction, and myelin depletion, and appear to be common factors in the onset of familial MS^19^. Considering the effect of MS individual's lifestyle, as well as the lack of diagnostic and prognostic biomarkers, it is important to identify therapeutic targets and proper biomarkers in MS condition. Our study focuses on the investigating the expression of *IL7R, NFATc2,* and *RNF213* pro-inflammatory genes. We also investigated their potential application in the diagnosis of the disease in the familial and sporadic MS patients versus healthy individuals with and without a family history of the disease. To investigate the effect of hereditary factors involved in the familial aggregation of the disease, we have divided patients into two different groups: familial and sporadic. Healthy first-degree relatives of the familial patients were also involved in the study to identify a distinct type of gene expression pattern that indicates a predisposition to MS.

## Materials and methods

### Study subjects

All patients were referred to Kashani hospital, affiliated with Isfahan University of Medical Sciences between June 2018 and September 2019, and blood samples afterwards were taken. The diagnosis of MS was based on the McDonald Criteria^20^. Blood samples were taken from 72 MS patients (35 sporadic, 37 familial), and 74 healthy individuals (34 healthy first-degree relatives of the familial MS patients (FDR), 40 healthy persons without a family history of MS (control)). All patients received IFN-Beta as a part of their treatment. Patients with coexistence of other neurological disorders were excluded from the study. The control group had no family history of the autoimmune or neurological disorders and were admitted for medical check-up. They were also sex/age-matched to the patient groups. All patients were evaluated for the extended disability status scale (EDSS) and were subjected to magnetic resonance imaging (MRI). The study was performed in accordance with the seventh edition of Helsinki declaration, approved by the ethics committee of the University of Isfahan, and informed consent was obtained from patients and healthy individuals. Table 1 presents more information on the clinical features of patients, and also demographic data of the healthy individuals.

**Table 1:** Clinical characteristics of the participants in this study.

Features		Sporadic patients N = 35	Familial patients N = 37	FDR group N = 34	Control group N = 40	p-value
Sex	Male	10 (28.6%)	9 (24.32%)	15 (44.11%)	11 (27.5%)	0.165
	Female	25 (71.4%)	28 (75.67%)	19 (55.88%)	29 (72.5%)	
Age		38.00 ± 10.59	36.26 ± 9.66	38.47 ± 9.28	37.38 ± 15.94	0.387
EDSS		2.11 ± 2.02	1.95 ± 2.27	–	–	0.541
Onset age		30.88 ± 10.13	28.97 ± 8.06	–	–	0.435
Time from first symptoms (years)		1.82 ± 3.92	3.30 ± 12.80	–	–	0.063
MS type	RRMS	19 (55.9%)	23 (71.9%)	–	–	0.037
	SPMS	5 (14.7%)	8 (25.0%)	–	–	
	PPMS	2 (5.9%)	0 (0.0%)	–	–	
	Other	8 (23.5%)	1 (3.1%)	–	–	
Consanguinity	Yes	6 (17.1%)	7 (29.2%)	–	–	0.274
	No	29 (82.9%)	17 (70.8%)	–	–	
Lesion load	Low	9 (28.1%)	5 (20.8%)	–	–	0.419
	Medium	13 (40.6%)	14 (58.3%)	–	–	
	High	10 (31.3%)	5 (20.8%)	–	–	
Cervical plaques	0	6 (19.4%)	4 (17.4%)	–	–	0.806
	1	8 (25.8%)	8 (34.8%)	–	–	
	1–3	7 (22.6%)	6 (26.1%)	–	–	
	> 3	10 (32.3%)	5 (21.7%)	–	–	

Values are mean ± SD or number (percent).

*EDSS* expended disability status scale, *SD* standard deviation, *RRMS* relapsing–remitting MS, *SPMS* secondary progressive MS, *PPMS* primary progressive MS, *BMI* body mass index.

### Demographic data of the participants

Our samples were classified into four different groups (35 sporadic patients, 37 familial patients, 34 FDRs, and 40 controls). In brief, 71.4% of the sporadic group (with an average age of 38.0 ± 10.5 years) and 75.6% of the familial group (with an average age of 36.2 ± 9.6 years) were female. Also, healthy participants were largely female (55.88%, 72.5%), with an average age of 38.4 ± 9.2, 37.3 ± 15.9 years in FDR and Control group, respectively. There was no significant difference in age (P = 0.38) and sex (P = 0.16) between separated groups. The Onset age and EDSS results were not altered significantly in sporadic and familial groups. Most of the patients in both groups were classified as relapsing–remitting type of MS (RRMS). The MRI results also were not meaningfully different in both patient groups. Table 1 presents the detailed characteristics of all patients and healthy groups.

### RNA isolation and reverse transcription

Total RNA was extracted from the peripheral blood of patients and healthy controls using the Favor Prep blood/cultured cell total RNA mini kit (Favorgen, Taiwan) according to the manufacturer’s protocol. The samples stored at − 70 °C. RNA concentration quantitated by Nano drop ND-100 (Thermo scientific, USA). Only samples containing completely pure RNA were used in the RT-PCR analysis, also the quality of this RNA samples was determined using electrophoresis. Reverse transcription was carried out in 10 μl reaction volume using a prime script RT reagent kit (Takara, Japan), following the standard instruction. The reaction mixture contained 0.5 μl of random hexamer primer, 0.5 μl of oligo dT primers, 2 μl of 5 × prime script buffer, 0.5 μl of prime script RT Enzyme Mix I, and 6.5 μl of extracting RNA solution plus RNase free water. The incubation program was as follows: 15 min at 37 °C, and 5 s at 85 °C. Finally, cDNA samples were stored at −20 °C.

### Quantitative real time-PCR

Target gene intron-spanning primers were designed by the NCBI online tool and oligo primer software (Bioneer Company), and primer sequences are presented in Table 2. Real time PCR was carried out on a Bio-Rad (Chromo4 TM, Bio-Rad Laboratories, USA) and also an ABI-7500 Real-Time PCR System (Applied Biosystems) using a Real Q plus 2 × master mix gene (Ampliqon, Odense, Denmark). Each PCR reaction was performed in a total volume of 10 μl, including 5 μl of the master mix, 0.5 μl reverse and 0.5 μl forward primer of target genes, and 4 μl of the reverse-transcribed cDNA. Reactions were run under standard conditions: initial denaturation at 95 °C for 15 min, 40 amplification cycles of 30 s denaturation at 95 °C and 30 s, annealing and elongation at 72 °C. The PCR products were separated on 1.5% agarose gel and produced the expected size amplicons for each specific target (Supplementary Fig. 1). The comparative Ct method (ΔCt) was used to estimate relative expression changes in our target genes. The expression levels were normalized to *ACTB* as a reference gene.

**Table 2:** Primer sequences used in qRT-PCR analysis.

Genes	Primers	Primer length	Product length	Tm	GC%
*B-ACTIN*	Forward: 5'TTCGAGCAAGAGATGGCCA3' Reverse: 5' CACAGGACTCCATGCCCAG3'	19 19	151	67/56 98/60	6/52 2/63
*IL7R*α	Forward: 5'TCATTCATTTCATACACACTGGCT3' Reverse: 5'TCCAAGTCTCCATTTTGAGCATAG3'	24 24	150	59/57 30/59	
*NFATc2*	Forward: 5'AACAAGCATATCCGCACACC3' Reverse: 5'GGTCATATTCATCCGTGGGCT3'	20 21	133	30/57 82/59	50 4/52
*RNF213*	Forward: 5'TGCTGGGAGACATGGAATGG3' Reverse: 5'GCCACACTGTCCTCTAAGCC3'	20 20	106	35/59 60/61	55 60

### Statistical analysis

The results were analyzed using the SPSS 26 software package (SPSS Inc. 2019. Chicago) and Graph Pad Prism 8 (Graph Pad Prism Software, Inc. San Diego CA, USA). Independent sample t-test was used to compare the differential expression between patients and control groups. Also the one-way ANOVA followed by LSD post hoc test was used to determine the statistical significance of expression differences among four analyzed groups.

We also investigated the correlation between *IL7R, NFATc2, RNF213* relative expressions and clinical variables, such as onset age and Expanded Disability Status Scale (EDSS). Spearman Correlation test were used to examine the association of relative gene expression levels with clinical parameters. The predictive power of the differentially expressed genes was calculated with plotting receiver-operating characteristic (ROC) curve (a graphical presentation of sensitivity versus 1-specificity) and also defining area under the curve (AUC). The AUC can be regarded as an indicator for evaluating the accuracy of a diagnostic test. The AUC value varies from 0 to 1, being close to 1 when the diagnostic assay has a high accuracy^21^. P ≤ 0.05 were considered statistically significant. The cutoff point is the threshold value of analytical signals below which the samples are negative and above which the samples are evaluated positive^22^. The optimal cut off point values for relative quantification that separates different groups was determined.

## Results

### Investigation of the relative gene expression levels of *IL7R*,* NFATc2*, and *RNF213* among genetically separated groups

**Figure 1:**
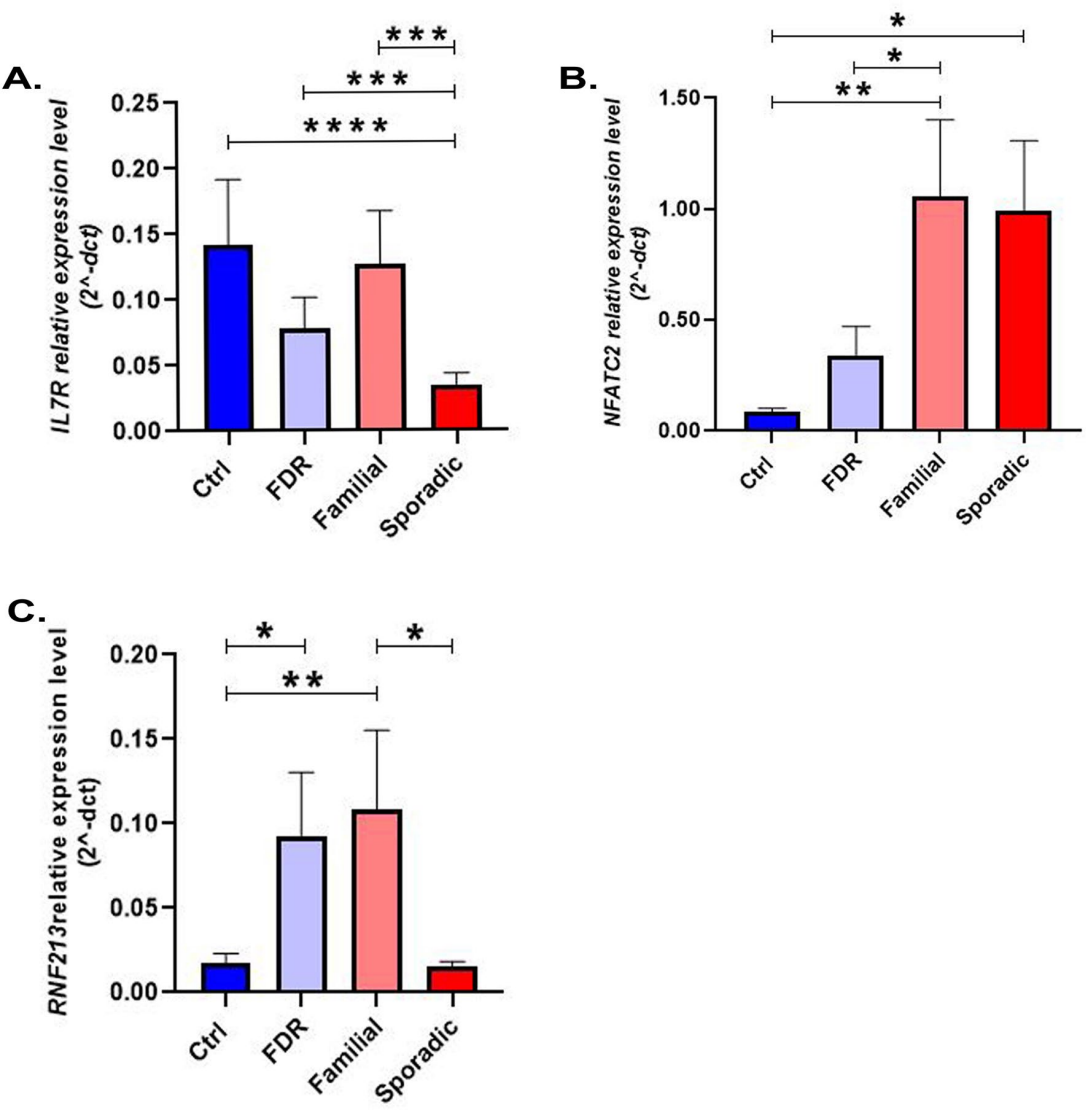
Differential expression levels of (**A)** *IL7R*, (**B)*** NFATc2* and (**C)*** RNF213* among genetically separated groups and control group. One-way ANOVA test and in the following, Fisher LSD post hoc test was used for the statistical analysis. *Ctrl* control group, *FDR* healthy first-degree relatives of the familial group. Bars represent the standard error of the mean. (*p < 0.05, **p < 0.01, ***p < 0.001).

We compared the expression level of *IL7R* in the four study groups. Our results demonstrated a significant down-regulation of *IL7R* in sporadic MS patients compared with the control group by 80%** ± **0.4515 (P_value_ < 0.0001) as well as the FDR group by 59% ± 0.4478 (P_value_ = 0.0005). There was also a significant decrease in the expression of sporadic versus familial patients by 70%** ± **0.4697 (P_value_ = 0.0004). We did not observe a significant difference in expression between the familial patients and healthy individuals (both Control and FDR) (Fig. 1A). Comparison of the *NFATc2* expression level in the four study groups demonstrated an increased expression in familial compared with control group by 314% ± 0.5975 (P_value_ = 0.0065), and also FDR group by 290% ± 0.6221(P_value_ = 0.031). There was also a significant increase in the expression of sporadic versus control group by 254% ± 0.6230 (P_value_ = 0.0148) (Fig. 1B). In the case of *RNF213* gene, the expression comparison among four groups demonstrated a similar gene expression level among familial and FDR groups. A significant increase in the expression of *RNF213* in familial by 277% ± 0.5420 (P_value_ = 0.0074), as well as the FDR by 227% ± 0.5621 (P_value_ = 0.0372) versus control group was also seen. The expression comparison demonstrated a significant increase in familial compared with sporadic patients by 225% ± 0.5592 (P_value_ = 0.038). A similar *RNF213* gene expression level among sporadic and control group was also seen (Fig. 1C).

### Investigation of the relative gene expression levels of *IL7R*,* NFATc2*, and *RNF213* among patients and the control group

**Figure 2:**
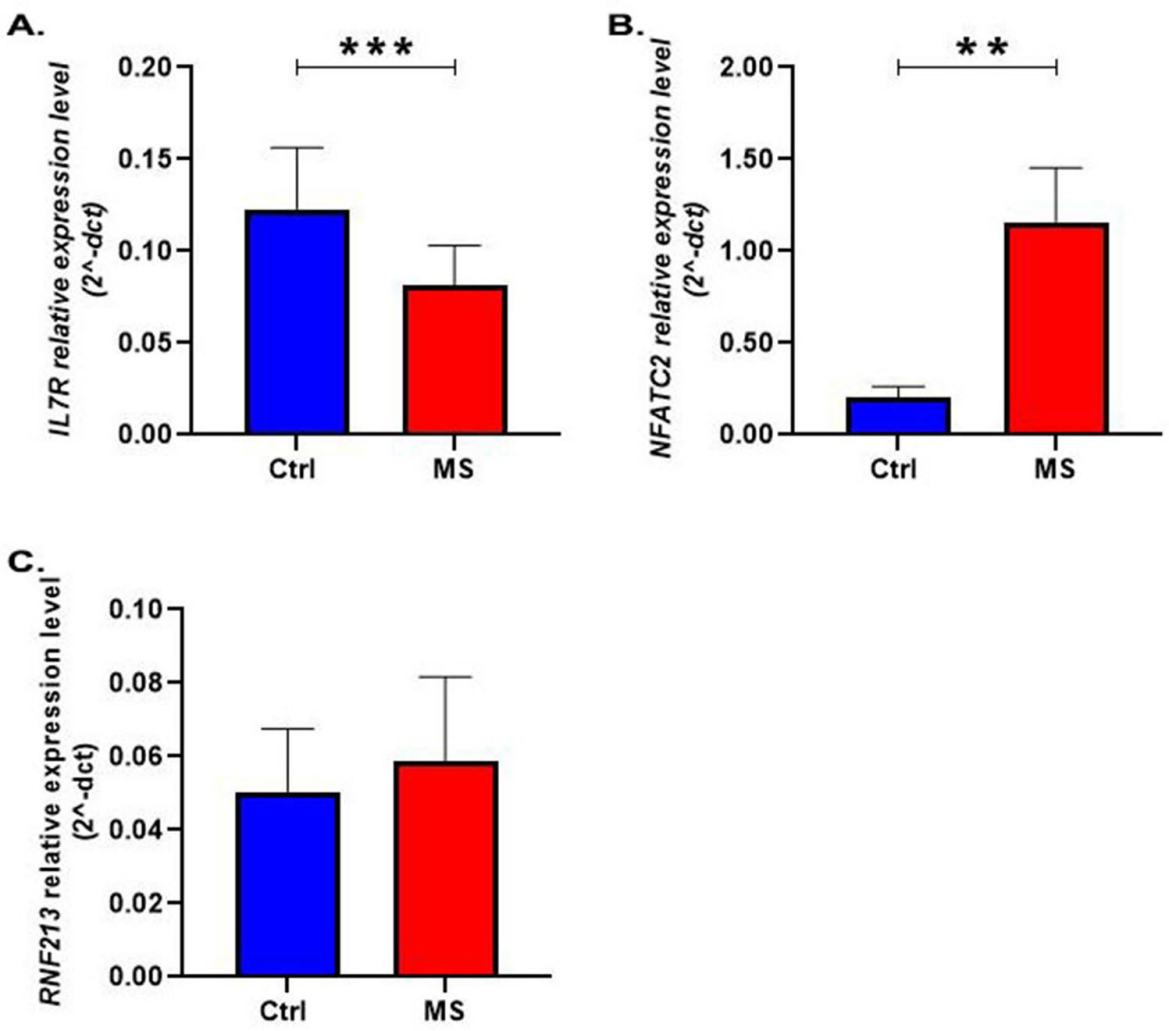
The relative expression level of (**A)** *IL7R*, (**B)** *NFATc2* and (**C)*** RNF213* in all MS patients (n:72) compared to healthy controls (n:74). *MS* multiple sclerosis, *Ctrl* control group. Bars represent the standard error of the mean. (*p < 0.05, **p < 0.01, ***p < 0.001) Unpaired t test with Welch’s correction and also Fisher LSD post hoc test was used for statistical analysis.

In this part of the study all sporadic and familial MS patients were classified as a single group: MS (n = 72). Also all healthy individuals were considered as a definite control group (n = 74). In this type of classification, the expression level of *IL7R* was lower in MS patients as compared to the control group by 58% ± 0.3586 (P_value_ = 0.0009) (Fig. 2A). Comparison of the *NFATc2* expression level in the two study groups showed an up-regulation in *NFATc2* mRNA level in the MS versus control groups by 275% ± 0.4586 (P_value_ = 0.0019) (Fig. 2B). No significant change in the *RNF213* expression level was observed between MS and control groups (P_value_ = 0.3550) (Fig. 2C).

### Diagnostic values of *IL7R*, *NFATc2* and *RNF213* among different study groups

**Figure 3:**
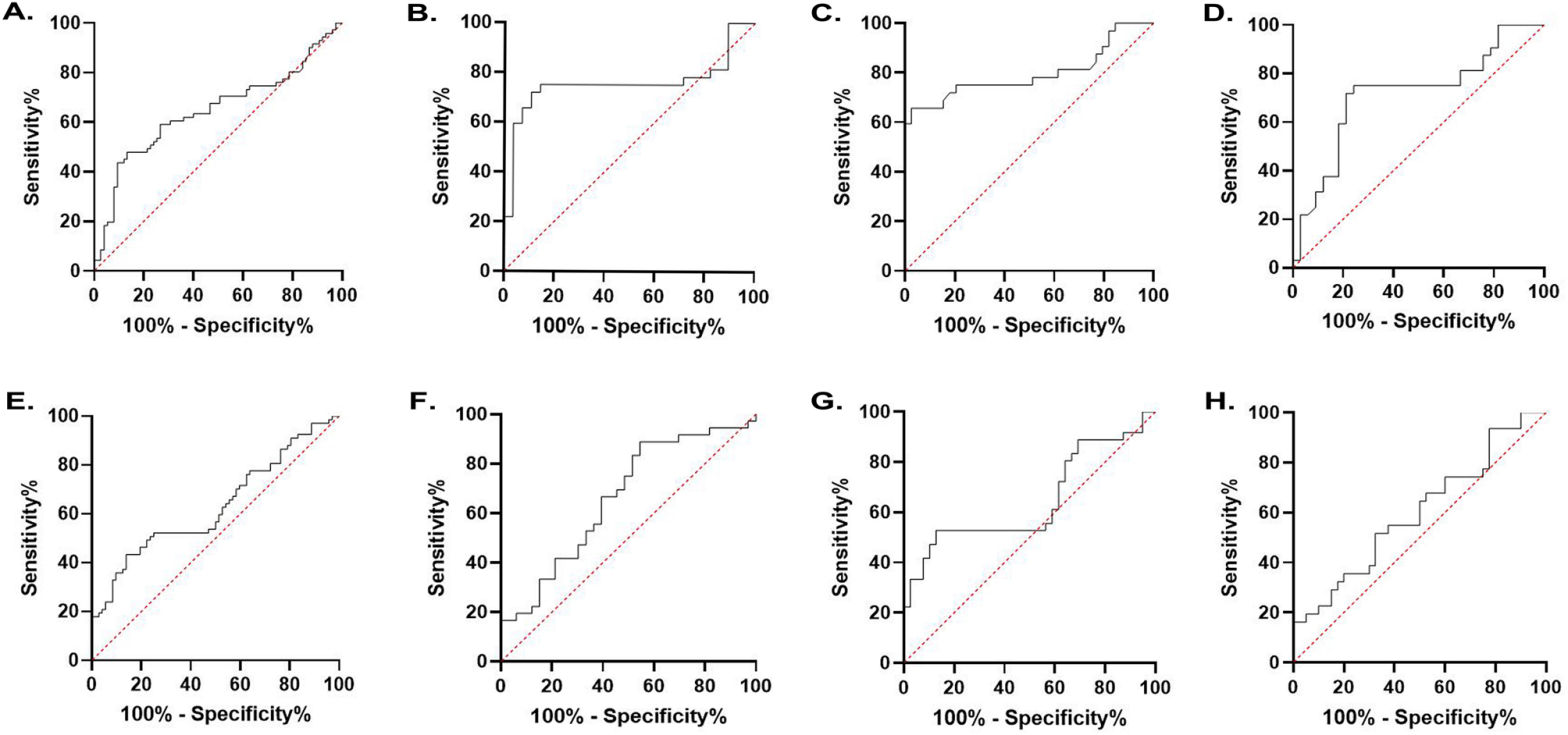
ROC curve analysis for determining statistically significant differences between study groups**. (A)** ROC curve of all patients and healthy controls analyzed for relative expression level of *IL7R* (AUC: 0.66, P_value_ = 0.0014). (**B**) ROC curve of sporadic patients and FDR groups analyzed for relative expression level of *IL7R* (AUC: 0.75, P_value_ = 0.0007). (**C)** ROC curve of sporadic patients and healthy controls analyzed for relative expression level of *IL7R* (AUC: 0.79, P_value_ < 0.0001). (**D**) ROC curve of sporadic patients and familial patients analyzed for relative expression level of *IL7R* (AUC: 0.71, P_value_ = 0.0030). (**E**) ROC curve of all patients and healthy controls analyzed for relative expression level of *NFATC2* (AUC: 0.63, P_value_ = 0.076). (**F)** ROC curve of Familial patients and FDR groups analyzed for relative expression level of *NFATC2* (AUC: 0.66, P_value_ = 0.021). (**G)** ROC curve of Familial patients and healthy controls analyzed for relative expression level of *NFATC2* (AUC: 0.64, P_value_ = 0.028). (**H)** ROC curve of Familial patients and healthy controls analyzed for relative expression level of *RNF213* (AUC: 0.67, P_value_ = 0.014).

Diagnostic test evaluation was performed for genes whose expression had changed significantly by ROC curve and AUC calculation. According to the expression level of *IL7R* between all patients and healthy controls, AUC was 0.66 (P_value_ = 0.0014) (Fig. 3A). Also, the optimal cut off value was defined as 0.0246 and the sensitivity and specificity values of 55.38% and 80.60% were achived, respectively. Plotting ROC curve for the sporadic patients versus FDR group (AUC = 0.75, P_value_ = 0.0007, cut off point = 0.019, sensitivity = 71.88%, specificity = 89.29%) (Fig. 3B), also sporadic patients versus healthy controls (AUC = 0.79, P_value_ < 0.0001, cut off point = 0.0202, sensitivity = 71.88%, specificity = 82.05%) (Fig. 3C), and sporadic patients versus familial patients (AUC = 0.71, P_value_ = 0.003, cut off point = 0.0201, sensitivity = 71.88%, specificity = 78.79%) (Fig. 3D), showed a significant result. AUC didn’t change significantly in other groups (Supplementary Fig. 2). In the diagnostic performance assessment of the *NFATc2* expression in MS versus the control group AUC was 0.66 (P_value_ = 0.0076) (Fig. 3E). Also, the optimal cut off value was defined as 0.0919 and the sensitivity and specificity values of 52.24% and 75.00% were achived, respectively. Plotting ROC curve for the familial versus FDR group (AUC = 0.66, P_value_ = 0.02, cut off point = 0.1181, sensitivity = 52.78%, specificity = 78.79%) (Fig. 3F), and also familial versus control group (AUC = 0.64, P_value_ = 0.028, cut off point = 0.01038, sensitivity = 52.78%, specificity = 87.18%) (Fig. 3G), showed a significant result. AUC didn’t change significantly in other groups (Supplementary Fig. 2). Diagnostic test evaluation was also performed for *RNF213* by ROC curve and AUC calculation. According to the expression level of *RNF213* in the familial versus control group, AUC was 0.67 (P_value_ = 0.014). Also, the optimal cut off value was defined as 0.0128, and the sensitivity and specificity values of 58.06% and 72.5% were achived, respectively (Fig. 3H). There was no statistical significance in other groups (Supplementary Fig. 2).

### Association of *IL7R*, *NFATc2* and *RNF213* gene expressions with clinical parameters

We investigated the association of *IL7R*, *NFATc2* and *RNF213* expression levels with EDSS and onset age in all MS patients and control groups, and separated four groups. The results did not show any significant correlation (Table 3).

**Table 3:** Correlation coefficients of relative gene expressions with EDSS and Onset age in patient groups.

Variable	Covariate	Sporadic patients	Familial patients	All patients
Current EDSS	*IL7R* relative expression (log2)	−0.220 (0.204)	−0.097 (0.596)	−0.157 (0.204)
	*NFATc2* relative expression (log2)	−0.130 (0.458)	0.013 (0.942)	−0.067 (0.588)
	*RNF213* relative expression (log2)	0.054 (0.757)	0.070 (0.701)	−0.064 (0.604)
Onset age	*IL7R* relative expression (log2)	0.043 (0.810)	0.188 (0.390)	0.003 (0.980)
	*NFATc2* relative expression (log2)	−0.272 (0.126)	0.057 (0.798)	−0.165 (0.224)
	*RNF213* relative expression (log2)	−0.102 (0.570)	0.701 (0.748)	−0.032 (0.815)

Bold values denote statistical significance at the p < 0.05 level.

## Discussion

Despite a great deal of research in the past, there is still no proper treatment for patients with MS, but researchers suggest a genetic effect on the etiology of the disease^23^. Previous Studies have investigated the factors involved in the familial aggregation of MS^24^. Due to the evidence indicating the association of *IL7R, NFATc2, and RNF213* with immunopathology of MS, in the present study, we analyzed their gene expression in familial and sporadic types of the disease and also healthy individuals to identify a distinct gene expression pattern that indicates a predisposition to the disease.

*IL7R* is a member of the type Ι cytokine receptor family. As mentioned before, the *IL7R*-*IL7R*α ligand-receptor pair play an important role in the survival and proliferation of B and T lymphocytes, and their genetic variations could lead to the immunodeficiency syndromes^12^. *IL7R* is located on chromosome 5pl3, and two promoter polymorphisms contribute to the genetic background of MS pathogenesis^25^. Previous studies have shown that *IL7R* can interact with Thymic Stromal Lymphopoietin (TSLP). TSLP is thought to trigger dendritic cell–mediated Th2-type inflammatory responses^26^. Th2 cells are believed to confer an anti-inflammatory response, and have been associated with decreasing inflammation^27^. Lei et al., have used metronidazole-induced demyelination in a transgenic zebrafish line in which oligodendrocytes expressed green fluorescent protein (GFP). Their results demonstrated a decrease in *IL7R* expression that induced JAK/STAT signaling pathway leading to oligodendrocytes apoptosis. These findings highlight the role of *IL7R* in the demyelination that is important in the pathogenesis of MS^28^, and increase the need to investigate the expression of *IL7R* in patients with MS. Therefore, interaction of *IL7R* with TSLP leads to the induction of T-helper 2 (Th2) cells^26^, which inhibits synthesis of a wide range of proinflammatory cytokines^27^. Also, according to the studies of the past that were mentioned, the decrease in *IL7R* expression caused demyelination^26,28^. Given that the role of this gene in MS is not clearly defined, we can expect a decrease in gene expression in MS patients. Of course, it should also be noted that gene expression can be different in various types of MS and people with different polymorphisms*.*

Regulatory T cells (Treg) express the transcription factor Foxp3 and show decreased expression of IL7Ra. Therefore the deficiency in suppression of autoimmune mechanisms may have an important role in multiple sclerosis pathogenesis^29^. In fact, previous studies have shown that single nucleotide polymorphisms (SNPs) in the *IL7R* and *IL2RA* may confer a risk in the susceptibility to MS. The identification of *IL7R* has made great strides in understanding the MS genetic risk factors^30^. Increasing evidence has shown that this gene also promotes oligodendrocyte survival and myelination^26^.

Our results showed that the *IL7R* was significantly decreased in the sporadic patients compared both to familial patients and to healthy controls. Since there is a significant differential expression between the sporadic and familial group, it is expected that familial patients do not show a significant difference in expression compared with FDR and control groups, and the results could directly confirm this .Additionally, the lower *IL7R* gene expression in all patients versus control groups might be significantly correlated with sporadic MS cases. The differential expression between the sporadic and familial groups can be attributed to SNPs in one of these groups, which in previous studies have shown in *IL7R,* that have affected the *IL7R* expression^29^. Investigation of the *IL7R* gene expression in MS patients in Australia, in 2005, showed a down-regulation in PPMS, and an up-regulation in SPMS compared to the controls^31^. In 2017, Bina et al. have evaluate the expression level and controlling role of *lnc-IL-7R* in the expression of two variants of *IL-7Ra* in MS patients versus healthy controls. they concluded no significant difference between the expression levels of *IL-7RB* and *IL-7RS* isoforms of *IL-7R* gene and *lnc-IL-7R* in MS patients (36 MS patient and 30 healthy controls)^32^. In 2018, a Bioinformatics study has identified miR-199a and miR-142–3-P as crucial biomarkers in MS due to targeting the pivotal susceptibility genes, in particular KRAS and *IL7R*. They also examined *IL7R* expression in a small population of MS and control patients (15 MS, 14 controls) and reported an increased expression of this gene, which contradicts our study^33^.

Wnt signaling pathway plays a key role in activating the myelination process as well as the differentiation and formation of oligodendrocytes^34^ . Therefore, it can be said that it is one of the key pathways in the MS disease. This pathway is also necessary for the maturation of the brain cells and is effective in reducing the penetration of immune cells. Besides, activation of Wnt/Ca^2+^ pathway regulates cytokine production in T cells^35^ .Previous studies have also suggested the role of the Wnt pathway in the development and oligodendrocytes re-myelination^36^.Therefore, this pathway can be targeted as a potential and effective treatment for MS patients. The Wnt/Ca^2+^ pathway activates the Nuclear Factor of Activating T-cell (NFAT)^37^. *NFATc2* is a member of the nuclear transcription factor activating a family of T cells, and is one of the Wnt/Ca^2+^ pathway genes, which translocates to the nucleus via dephosphorylation by calcineurin. As a result, it activates the Wnt/Ca^2+^ pathway target genes^38^. In 2019, Vilariño-Güell et al. performed whole-exome sequencing analysis in 132 patients from 34 families. In this study, pathogenic variants for MS in 12 genes of the innate immune system that regulate the transcription and activation of inflammatory mediators were nominated. *NFATc2* and *RNF213* were among these genes and provide the molecular and biological rationale for the inflammation, demyelination and neurodegeneration observed in MS patients. In this experiment, *NFATc2* knockout mice indicated an increase in immune responses, as well as the production of cytokines in T cells^19^. The induction of EAE in *NFATc2* knockout mice causes a markedly reduced clinical score, compared to wild-type animals^39^. According to these studies, it seems that *NFATc2* suppression can be considered as a potential disease-modifying treatment for MS patients. Thus, it would be expected that the expression level of *NFATc2* is increased in MS patients compared with controls. In the present study, the relative expression level of *NFATc2* is significantly up-regulated in sporadic and also familial groups, compared with healthy controls. Additionally, we observed significant differences in the of *NFATc2* expression level among familial patients and FDR. The results of our study indicated that the expression of *NFATc2* is not significantly different between sporadic and familial patients, and therefore the *NFATc2* can not act as a biomarker in distinguishing familial and sporadic types. However, since *NFATc2* expression in familial patients has changed significantly compared with their first-degree relatives, this gene can be considered as a possible prognostic marker in affected families. This hypothesis is acceptable if FDR is no longer supposed to develop the disease. Thus, down regulation of the *NFATc2* in FDR versus familial can be introduced as a prognostic biomarker. Since we preferred older healthy first-degree relatives, the possibility of using this gene as a prognostic biomarker could be strengthened. However, more studies are needed to prove this hypothesis. As expected, *NFATC2* mRNA level was also up-regulated in all MS patients versus all controls.

*RNF213* is located on chromosome 17 and is one of the genes involved in the inflammatory pathways, such as Wnt/Ca^2+^. This gene is expressed in most tissues of the body, but its physiological function is still much unknown. *RNF213* encodes a unique, 591-KDa protein with both a ring finger domain and walker motifs^40^. Ring-based E_3_ ligases have been linked to the control of many cellular processes, including proteasome-dependent proteolysis, immunological processes and transcription^41^. Therefore, *RNF213* is an ATPase and E_3_ ubiquitin ligase protein. *RNF213* also targets *NFATc2* proteasomal degradation and attenuats the non-canonical Wnt/Ca^2+^ pathway. As a result, *RNF213* deficiency has been shown to trigger an increased expression of NFAT target genes^19^. Thus, because the expression of the *NFATC2* increased in the pathways leading to MS, the expression of the *RNF213* gene is expected to decrease. In the present study, a similar *RNF213* gene expression level among familial and FDR groups was seen, but gene expression in both groups had significantly increased compared to the control group. Based on the observed results, the differential expression of this gene can be considered as a predisposing factor in FDR individuals. Most of these individuals may remain asymptomatic for the rest of their lives or develop the disease due to different environmental factors. It seems that the existence of a specific variant in all family individuals could lead to an increased gene expression, compared with healthy individuals without a family background. Also different miRNAs have the potential to affect the expression of a gene. Therefore, it is also possible that the *RNF213* expression in familial individuals has been affected in this way. Additionally, the expression comparison demonstrated a significant increase in familial compared with sporadic patients and a similar *RNF213* gene expression level among sporadic and control group was seen. Therefore, it is concluded that only familial cases show a significant change in expression of this gene, and *RNF213* may not be considered as a biomarker in sporadic MS cases. The averaged *RNF213* expression is similar for MS and control groups. Here, by averaging all patients real differences are masked, resulting in the wrong conclusion, and misinterpretation of the data. But we prefere to provide these results to show the need to classify patients into sporadic and familial categories in the future studies. However, since no studies have been found to investigate the expression level of *RNF213* in MS versus healthy individuals, the results of the study are not comparable. It seems that by examining this gene in different populations and in a larger statistical population, more reliable results will be achieved. Additionally, we could find a study that investigated the association of *RNF213* with MS pathogenesis, which was conducted in 2021. In this study, multiple variants in several genes, including *RNF213*, were introduced as an effective factor in the development of MS^42^.

This research is the first study to examine the expression of *IL7R, NFATc2* and *RNF213* in MS patients versus healthy controls. Also, it is the first study that has evaluated the expression level of *IL7R*, *NFATc2* and *RNF213* among MS groups with genetic classification, to investigate the effect of hereditary factors involved in the familial aggregation of the disease. However, this research also has some limitations, which can be pointed out by the small statistical population of the participants. Further studies in a larger group are necessary to determine whether *IL7R*, *NFATc2* and *RNF213* can be used as biomarkers for the prognosis of MS.

## Conclusions

Today, many genetic tests use prognostic biomarkers to predict disease before clinical manifestation, to inform family members at risk. In this study, an attempt was made to help the prognosis of people at risk. Therefore, this research has investigated the potential role of *IL7R*, *NFATc2* and *RNF213* as prognostic markers in MS patients. According to the results of our study, the down regulation of *IL7R* can be considered as a biomarker for sporadic but not familial MS patients. In the case of *NFATc2*, there is no difference between controls, nor between MS groups. Therefore, its upregulation is associated with the disease in both familial and sporadic MS. Additionally, only familial cases show a significant change in *RNF213* expression. But, since there is no difference between the diseased and healthy relatives, it couldn't be used as a prognostic biomarker in MS cases.

The results of our study will improve the development of appropriate treatment plans for sporadic and familial MS patients separately and plays a significant role in the introduction of potential prognostic markers. Also, in the future, similar studies will have interesting implications in personalizing the treatments.

## Supplementary Information

Supplementary Figures.
