# Supplementary material for: Expression and clinical significance of IL7R, NFATc2, and RNF213 in familial and sporadic multiple sclerosis
Source: Sci Rep. 2021 Sep 28;11:19260. doi: 10.1038/s41598-021-98691-5 (PMC8478940; doi:10.1038/s41598-021-98691-5)
Supplement: Supplementary file 1 — Supplementary Figures. [file 41598_2021_98691_MOESM1_ESM.pdf]

Expression and clinical significance of *IL7R*, *NFATc2* and *RNF213* in familial and sporadic multiple sclerosis patients

Seyedeh Zahra Hosseini Imani[1], Zohreh Hojati[1*], Sheyda Khalilian[1], Fariba Dehghanian[1], Majid Kheirollahi[2], Mehdi Khorrami[2], Vahid Shaygannejad[3], Omid Mirmosayyeb[3]

1. Division of Genetics, Department of Cell and Molecular Biology and Microbiology, Faculty of Biological Sciences and Technologies, University of Isfahan, Isfahan, Iran.
2. Research Institute for Primordial Prevention of Non-Communicable Disease and Department of Genetics and Molecular Biology, School of Medicine, Isfahan University of Medical Sciences, Isfahan, Iran.
3. Isfahan Neuroscience Research Center, Isfahan University of Medical Sciences, Isfahan, Iran.

* Correspondence: Dr. Zohreh Hojati

Postal Code: 81746-73441

Email: z.hojati@sci.ui.ac.ir

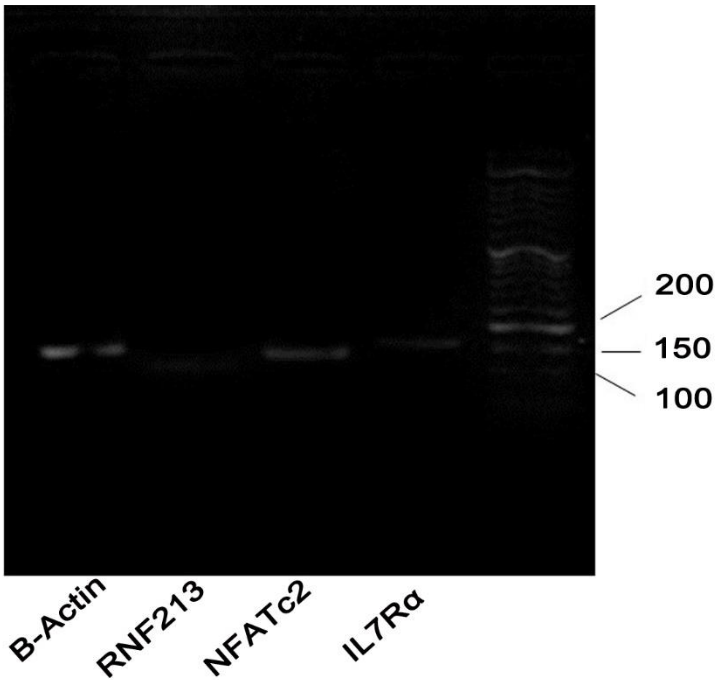

Supplementary Fig. 1 DNA gel electrophoresis for validation of gene amplification in RT-PCR. A patient sample run on a 2% agarose gel stained with Ethidium Bromide. The first line accommodates a 50 base bp ladder. Amplicon size of *IL7R* = 150 bp, *NFATc2* = 133 bp, *RNF213* = 106 bp, *ACTB* =151 bp

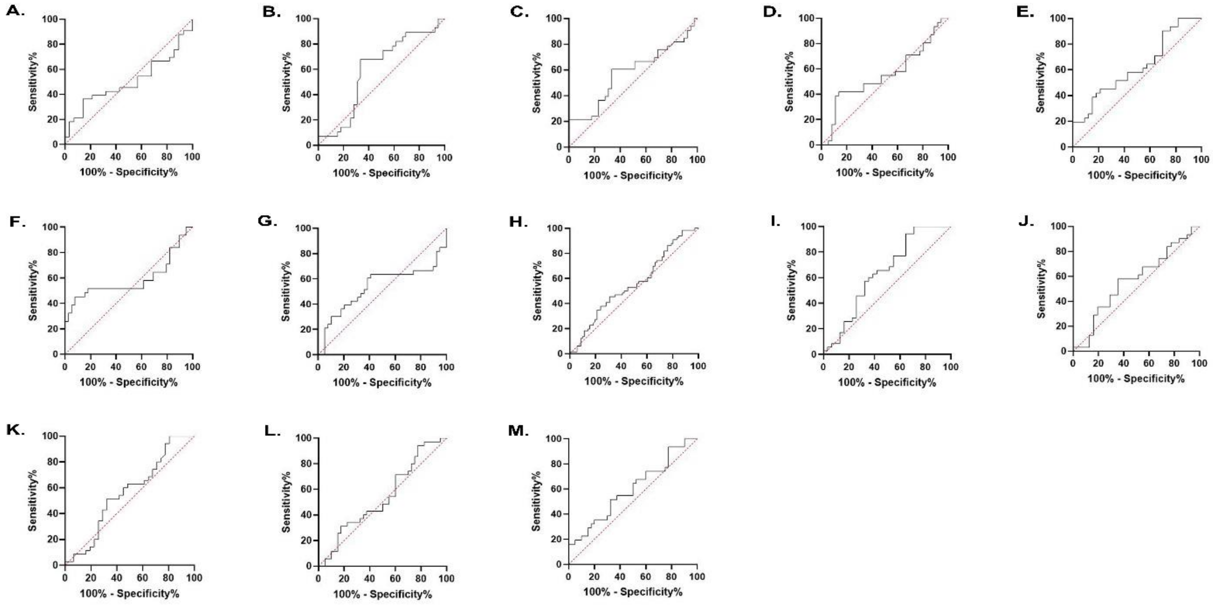

Supplementary Fig. 2 ROC curves for *IL7R*, *NFATc2* and *RNF213*. **A)** ROC curve of FDR and Familial patients analyzed for the relative expression level of *IL7R* (AUC: 0.504, p value: 0.95). **B)** ROC curve of FDR and healthy controls analyzed for relative expression level of *IL7R* (AUC: 0.59, p value: 0.17). **C)** ROC curve of Familial patients and healthy controls analyzed for relative expression level of *IL7R* (AUC: 0.57, p value: 0.26). **D)** ROC curve of Sporadic and Familial patients analyzed for relative expression level of *NFATC2* (AUC: 0.55, p value: 0.47). **E)** ROC curve of Sporadic patients and FDR analyzed for relative expression level of *NFATC2* (AUC: 0.62, p value: 0.09). **F)** ROC curve of Sporadic patients and healthy controls analyzed for relative expression level of *NFATC2* (AUC: 0.58, p value: 0.19).**G)** ROC curve of FDR and healthy controls analyzed for relative expression level of *NFATC2* (AUC: 0.53, p value: 0.59). **H)** ROC curve of MS patients and controls analyzed for relative expression level of *RNF213* (AUC: 0.55, p value: 0.25). **I)** ROC curve of Familial and Sporadic patients analyzed for relative expression level of *RNF213* (AUC: 0.63, p value: 0.053). **J)** ROC curve of Familial patients and FDR analyzed for relative expression level of *RNF213* (AUC: 0.56, p value: 0.37). **K)** ROC curve of Sporadic patients and FDR analyzed for relative expression level of *RNF213* (AUC: 0.56, p value: 0.38). **L)** ROC curve of Sporadic patients and healthy controls analyzed for relative expression level of *RNF213* (AUC: 0.53, p value: 0.55). **M)** ROC curve of FDR and healthy controls analyzed for relative expression level of *RNF213* (AUC: 0.59, p value: 0.15).
